# Heat Shock protein 90: Role in Enterovirus 71 Entry and Assembly and Potential Target for Therapy

**DOI:** 10.1371/journal.pone.0077133

**Published:** 2013-10-02

**Authors:** Yueh-Liang Tsou, Yi-Wen Lin, Hsuen-Wen Chang, Hsiang-Yin Lin, Hsiao-Yun Shao, Shu-Ling Yu, Chia-Chyi Liu, Ebenezer Chitra, Charles Sia, Yen-Hung Chow

**Affiliations:** 1 Institute of Infectious Disease and Vaccinology, National Health Research Institutes, Zhunan Town, Miaoli, County, Taiwan; 2 Graduate Program of Biotechnology in Medicine, Institute of Molecular and Cellular Biology, National Tsing Hua University, Hsinchu, Taiwan; 3 Graduate Institute of Immunology, China Medical University, Taichung, Taiwan; University of Malaya, Malaysia

## Abstract

Although several factors participating in enterovirus 71 (EV71) entry and replication had been reported, the precise mechanisms associated with these events are far from clear. In the present study, we showed that heat shock protein 90 (HSP90) is a key element associated with EV71 entry and replication in a human rhabdomyosarcoma of RD cells. Inhibition of HSP90 by pretreating host cells with HSP90β siRNA or blocking HSP90 with a HSP90-specific antibody or geldanamycin (GA), a specific inhibitor of HSP90, as well as recombinant HSP90β resulted in inhibiting viral entry and subsequent viral replication. Co-immunprecipitation of EV71 with recombinant HSP90β and colocalization of EV71-HSP90 in the cells demonstrated that HSP90 was physically associated with EV71 particles. HSP90 seems to mediate EV71 replication by preventing proteosomal degradation of the newly synthesized capsid proteins, but does not facilitate viral gene expression at transcriptional level. This was evident by post-treatment of host cells with GA, which did not affect the expression of viral transcripts but accelerated the degradation of viral capsid proteins and interfered with the formation of assembled virions. *In vivo* studies were carried out using human SCARB2-transgenic mice to evaluate the protection conferred by HSP90 inhibitor, 17-allyamino-17-demethoxygeldanamycin (17-AAG), an analog of geldanamycin, that elicited similar activity but with less toxicity. The results showed that the administration of 17-AAG twice conferred the resistance to hSCARB2 mice challenged with C2, C4, and B4 genotypes of EV71. Our data supports HSP90 plays an important role in EV71 infection. Targeting of HSP90 with clinically available drugs might provide a feasible therapeutic approach to treat EV71 infection.

## Introduction

Enterovirus 71 (EV71) is a single-stranded RNA virus belonging to the *Picornaviridae* family. EV71 is associated with HFMD and even severe neurological disorders, including encephalitis, acute flaccid paralysis, pulmonary edema (PE), or hemorrhage, culminating in fatality, particularly in children under five years [1–5]. Although the emerging EV71 infection could potentially become a new threat to global public health [1,6–11], effective anti-viral drugs and a prophylactic vaccine are under development. Knowledge of cellular proteins participating in EV71 infection would facilitate an understanding of virus-host interactions and help identify crucial molecular targets for development of antiviral drugs.

Numerous animal models had been developed to serve as EV71 infectious models. Animal models using newborn (1-d- to 1-wk-old) but not older ICR or BALB/c mice only showed neurological pathology but no HFMD syndrome when infected with the natural non-existing mouse-adapted EV71 [12–17], or with natural strains of EV71 in type I/II interferon-deficient newborn mice [18] or in cynomolgus monkeys [19]. These are not perfect models for HFMD resembling neuropathogenesis caused by EV71 in humans due to narrower time window allowing for EV71 induced disease, and the limitations of experimental manipulations in monkey model. Recently, two receptors of EV71, human P-selectin glycoprotein ligand-1 (hPSGL-1 [20]) and human scavenger receptor class B, member 2 (hSCARB2 [21]); have been discovered. Taking advantage of these findings, we had generated transgenic mice expressing Human SCARB2 (hSCARB2-Tg) and proved that hSCARB2-Tg mice have greater susceptibility and pathogenesis, induce both HFMD and neurological diseases upon infection with EV71 isolates of genotype B and C [22]. Human PSGL-1 transgenic mice were also generated but failed to enhance the diseases of clinical EV71 strains [23].

Besides hSCARB2 and hPSGL-1, other cellular proteins that are involved in EV71 infection have been identified. An adherent factor of annexin II interacts with EV71 *via* VP1 binding and enhances viral infectivity [24]. Cell surface heparan sulfate plays as an attachment receptor for EV-71 infection [25]. Sialic acid-linked O-glycans and glycolipids, but not N-glycans, supports EV71 infection [26]. Heterogeneous nuclear ribonuclear protein K binds to 5’ untranslated region of EV71 and participates in virus replication [27]. A positive internal ribosome entry site (IRES) trans-acting factor, far upstream element binding protein 1, binds to IRES of EV71 and subsequently enhances viral translation in infected cells [28]. In this paper, we report that Heat shock protein 90 (HSP90) is involved in EV71 infection and might serve as a target for the development of anti-EV71 medications. Heat shock proteins are the products of several distinct gene families that are required for cell survival during stress and are named according to the approximate relative molecular mass of their encoded proteins including HSP10, HSP27, HSP40, HSP60, HSP70, HSP90, and HSP110 [29]. HSP90 is a chaperone interacting with a wide variety of important target proteins for cell signaling and regulation during tumorgenesis [30,31]. HSPs bind to unfolded sequences of newly synthesized polypeptides when the cell is under stress and form complexes of chaperones that mediate primary polypeptides to fold to form an adequate tertiary structure of the functional protein [32,33]. After completion of their chaperone function, HSPs are actively released from protein substrates by means of their intrinsic ATPase domain [34]. It was reported that HSP90 as an essential factor for folding and maturation of picornavirus capsid proteins [35]. Therefore, we hypothesized that EV71 infection may induce and use HSP90 for its replication. We demonstrated that HSP90 directly interacted with EV71 on the cell surface. Down-regulation of cellular HSP90 by specific inhibitors such as geldanamycin (GA), siRNA or antibody, all interfered the early stage of viral entry as well as viral replication and the stability of viral capsid proteins was impaired at the post-translational level. Subsequently, newly synthesized EV71 capsid proteins were not able to assemble properly in the infected cell. *In vivo* therapy with a less toxic drug, a GA analog,17-allyamino-17-demethoxygeldanamycin (17-AAG), in hSCARB2-Tg mice prior to challenge with EV71 isolates, 5746-TW98 (C2), N-3340 (C4) or E59 (B4), resulted in the survival of 100%, the relief of hind limb paralysis, or the inhibition of HFMD-like disease of mice compared to the control group without drug treatment. These results reveal the role of HSP90 in EV71 infection and the potential of treating EV71 patients with anti-HSP90 inhibitors.

## Results

### Knocking down HSP90 inhibits EV71 infection

**Figure 1:**
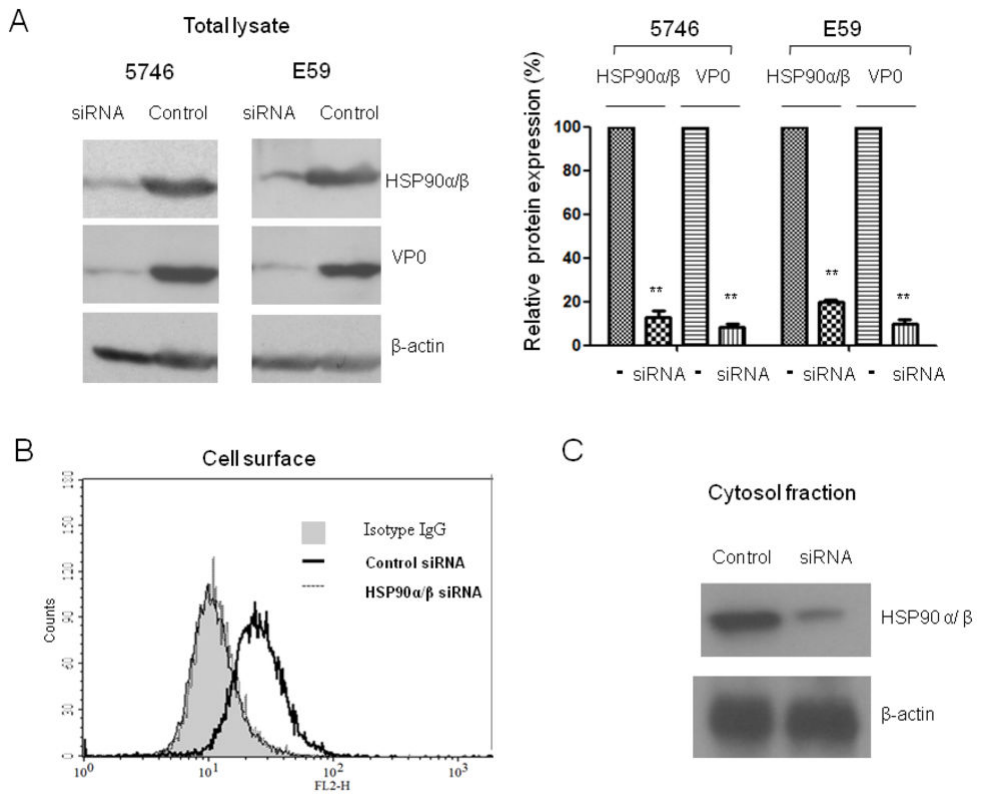
Down-regulation of HSP90α/β inhibits EV71 infection. 200 pmoles of siRNA specific to HSP90 or the same amounts of control siRNA were transfected to RD cells. After 24 hours of transfection, cells were following infected separately with MOI=0.01 of C2 and B4 genotypes of EV71 5746 and E59 strains. Cells were washed after one hour of infection and cultured for another 16 hours. (A) Cells were lysed and detected the content of HSP90α/β and EV71 capsid proteins by Western blot using anti-HSP90α/β and Mab979 antibodies, respectively. Cellular β-actin was detected by blotting the same membrane with monoclonal anti-β-actin antibody. Corresponding changes in HSP90α/β and EV71 capsid proteins levels compared to the expression levels in the cells with 200 pmoles control siRNA as 1.0 were quantified using Image-Pro Plus 6.0 software. (B) Cells were incubated with anti-HSP90α/β antibody (H-114) or control rabbit IgG (filled curve) at 4°C for another one hour. The cells were washed and fixed by 2% paraformaldehyde in PBS for 30 min. After washing, the treated cells were incubated with anti-rabbit IgG-TR antibody. Stained cells were run on a FACScan flow cytometer and analyzed by using CellQuest software. (C) Cytosolic fractions were prepared as described in the materials and methods and subjected to western blot to detect HSP90α/β as described in (A). Data represent one of two independent experiments. Unpaired student *t* test with Welch correction was used for statistical analysis (*p<=0.05, **p<=0.01).

To prove that HSP90 is the key protein supporting EV71 infection, endogenous HSP90 expressed in RD cells was knocked down using specific siRNA. Cells were transfected with HSP90 siRNA and subsequently infected with B4 genotype EV71 E59 or C2 genotype of EV71 5746 strains. Multiple EV71 capsid proteins are generated from P1 primary translates which encoded from the transcript products of P1 open reading frame processed by viral protease 3CD, VP0 (38 kDa, a precursor product of VP2 + VP4), VP1 (36 kDa), VP2 (28 kDa), VP3 (25 kDa) and VP4 (8 kDa) were detected in the EV71-infected cells [36–38]. Expression level of HSP90α/β and EV71 capsid proteins in siRNA-transfected cells was monitored by Western blot using anti-HSP90α/β antibody (N-17) and Mab979 antibody specific to EV71 capsid proteins, respectively. The relative expression of the target proteins was compared by quantification of the intensity obtained from the protein signal in the gel. Our results showed that the expression of HSP90 was reduced to 15% in RD cells transfected with HSP90α/β siRNA which targeted the conserved sequence of HSP90α (nucleotide 772-794, Accession number: HSP90AA1) and HSP90β (nucleotide 297-319, Accession number: HSP90AB1) mRNA, compared to control siRNA-transfected cells (100% of relative expression; Figure 1A). We also measured the levels of HSP90 on the surface by flow cytometry and the cytosolic HSP90 by Western blot, respectively. The significant downregulation of HSP90 in both sites of cells treated with HSP90α/β siRNA, compared to control siRNA, was observed (Figure 1B and 1C). Inhibition of total HSP90 expression by the siRNA significantly reduced the expression of EV71 E59 and 5746 capsid protein VP0 in the infected cells, the expression of VP0 was not affected in mock-transfected cells (Figure 1A).

**Figure 2:**
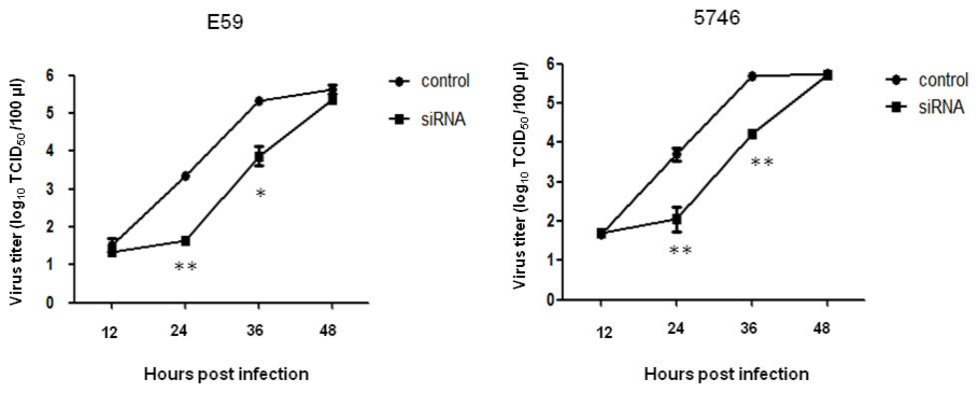
HSP90α/β siRNA inhibits EV71 production. 200 pmoles of HSP90α/β siRNA or the same amounts of control siRNA were transfected to RD cells and cultured for 24 hours, Cells were following infected separately with MOI=0.01 of EV71 5746 and E59 strains. Cells were washed after one hour of infection and cultured for another 12, 24, 36, and 48 hours. After cultivation, the supernatants were harvested and subjected to assay virus titer (TCID_50_) as described in the Materials and Methods. Data represented one of two independent experiments. Unpaired student *t* test with Welch correction was calculated.

To examine whether the EV71 production was affected by the downregulation of HSP90 expression in RD cells, the kinetic of virus titer (TCID_50_) in the medium of cells treated with HSP90α/β siRNA or control siRNA was assayed. Significant inhibition of both strains of EV71 production at 24 or 36 hours post infection were observed in the cells pretreated with HSP90α/β siRNA compared to the control siRNA. No difference at the late 48 hours post infection was observed, probably due to the newly synthesized HSP90 in the cell was begun (Figure 2). These results confirm a functional relation between HSP90 and EV71 infection.

### Down-regulation of HSP90β. but not HSP90α, inhibits EV71 infection

**Figure 3:**
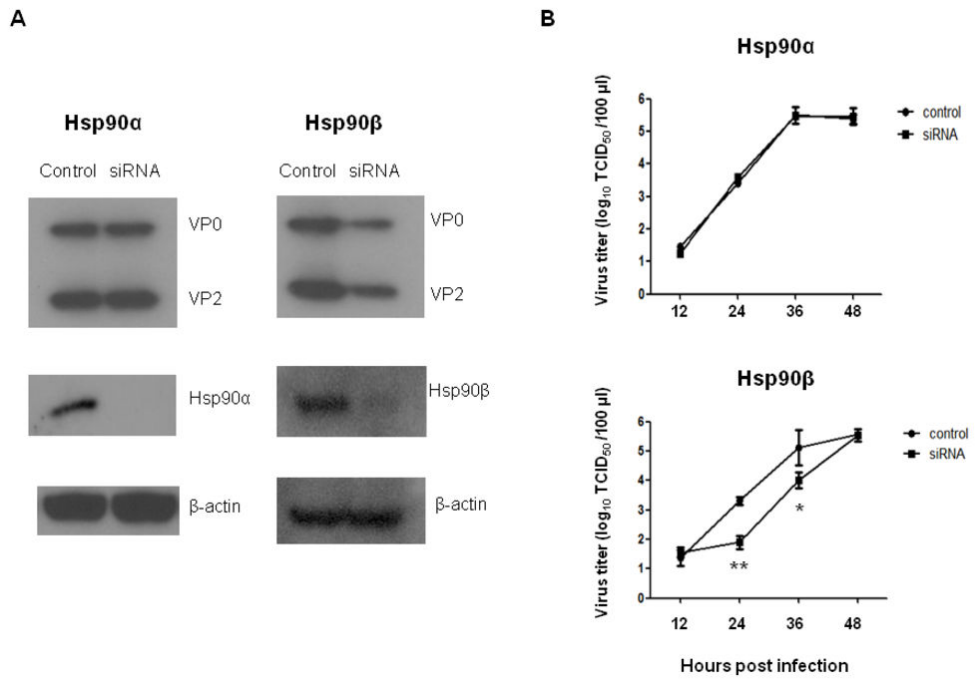
HSP90β but not HSP90α siRNA inhibits EV71 infection. RD cells were transfected individually with 200 pmoles of HSP90α, HSP90β, or the control siRNA, and then incubated for 24 hours, Cells were following infected with MOI=0.01 of EV71 5746 strain. Cells were washed after one hour of infection and cultured for another 16 hours. (A) Cells were lysed and detected the EV71 capsid proteins, HSP90α, and HSP90β by Western blot using specific antibodies, respectively. Cellular β-actin was detected by blotting the same membrane with monoclonal anti-β-actin antibody. (B) The culture mediums were harvested at 12, 24, 36, and 48 hours post infection and applied to detect virus titer (TCID_50_) as described in the Materials and Methods. Data represented one of two independent experiments. Unpaired student *t* test with Welch correction was calculated.

Because Hsp90 chaperon formed as a homodimer and/or heterodimer by HSP90α and HSP90β isoforms is required for its function (Minami et al. 1994). We further addressed which isoform of HSP90 participated in EV71 infection. RD cells were transfected with HSP90α- or HSP90β-specific siRNA for 24 hrs prior to EV71 5746 infection. The protein expression of HSP90α and HSP90β was all reduced by their specific siRNA, compared to control siRNA (Figure 3A). However, only HSP90β-specific siRNA abrogated EV71 capsid protein expression (Figure 3A). Similar scenarios were seen in the inhibition of EV71 production by HSP90β- but not HSP90α-specific siRNA (Figure 3B) and confirmed that HSP90β participated in EV71 infection.

### Inhibition of EV71 entry by HSP90 inhibitor

**Figure 4:**
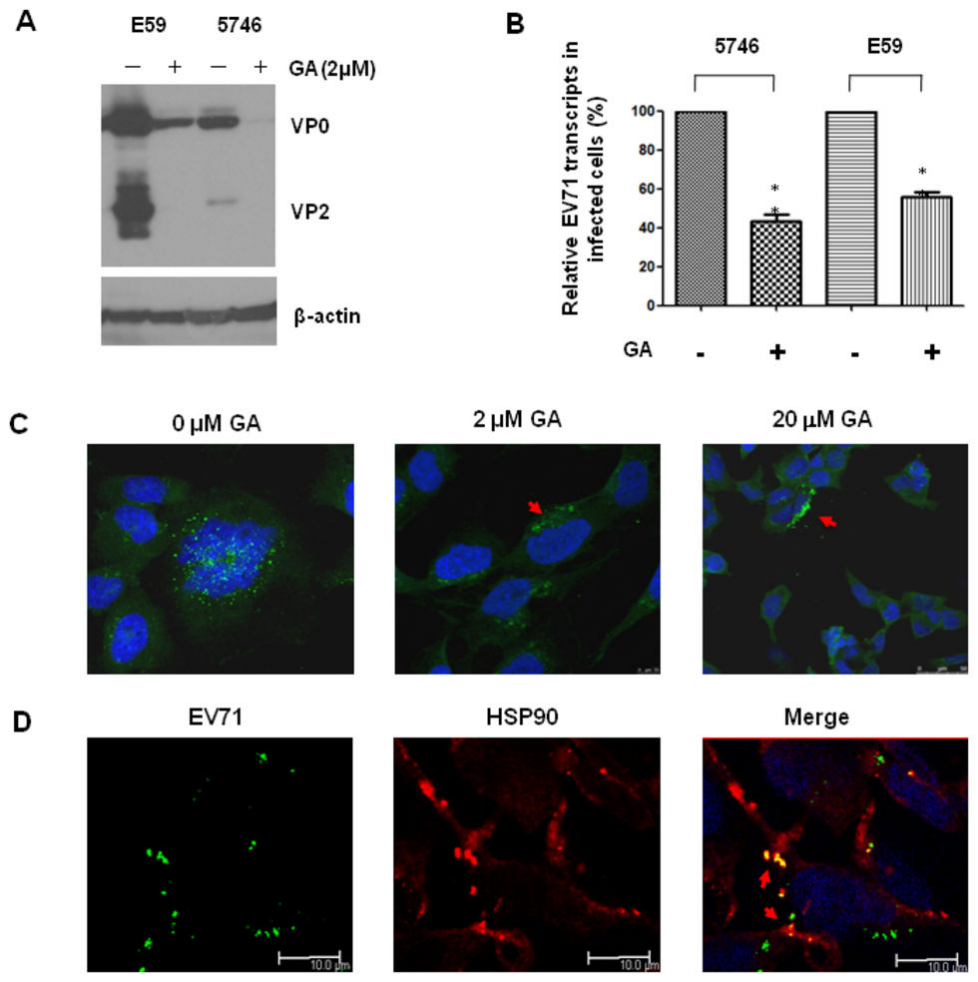
Inhibition of EV71 entry by pretreatment with HSP90 inhibitor. Pretreatment of RD cells with 2 µM or 0 µM (0.1% DMSO) of GA for one hour before MOI = 0.01 of EV71 5746 (C2) and E59 (B4) infection was conducted individually. After one hour infection, (A) cells were washed with Opti-MEM medium and incubated for 16 hours. Cells lysate were further prepared to detect EV71 capsid protein by western blot using MAB979 antibody. The internal cellular β-actin was detected by blotting the same membrane with monoclonal anti-β-actin antibody. (B) RNA was extracted at 90 min post infection and subjected to quantify EV71 transcripts by real-time RT-PCR using the primers against VP1 region. The relative expression of target gene was calculated as described in the Materials and Methods. Each normalized 2^Ct^ value from GA-treated cells was ratio to the value from the mean of 2^Ct^ obtained from the vehicle-treated cells. A schematic representation of the EV71 transcripts expression was shown. Data represented one of two independent experiments. Unpaired student *t* test with Welch correction was used for statistical analysis. (C) RD cells were treated with various concentration of GA or 0 µm of GA at 37°C for one hour before EV71 (MOI=20) infection for another one hour. After infection, cells were fixed before antibody staining. EV71 was stained with Mab979 antibody. (D) RD cells infected with EV71 (MOI=20) at 4°C for one hour without GA treatment were fixed and stained with Mab979 and anti-HSP90 antibodies (N-17). The respective secondary antibodies conjugated with fluorescence dye described in the Materials and Methods were used. Detection of EV71 (green), HSP90 (red), EV71-HSP90 merging (yellow) and nuclei stained with DAPI (blue) using a confocal microscope Leica TCS SP5 II was performed. The pictures were taken at 200x magnification from 2 independent experiments and one of them was shown.

In an attempt to unravel the mechanism of HSP90 involved in EV71 infection, a naturally existing HSP90 inhibitor, geldanamycin (GA) that belongs to the benzoquinone ansamycin family, was used. GA binds to the amino terminal ATP-binding pocket of HSP90 and inhibits ATP binding and hydrolysis. The binding of GA to HSP90 interferes with HSP-mediated target protein folding, leading to target aggregation and degradation [30,31,39]. To confirm the role of HSP90 in EV71 entry, RD cells were pretreated with GA or vehicle (0.1% DMSO) before infection with EV71 E59 or 5746 strains. Pretreatment of GA (up to 20 µM) in RD cells for 3 and 24 hours was shown no cytotoxic effect (Figure S1). The levels of EV71 capsid proteins VP0 and VP2 in 2 µM GA-treated cells were detected by Western blot (Figure 4A) and viral transcripts in the cells were quantified by real-time RT-PCR (Figure 4B). Significant inhibition of both EV71 isolates was observed in protein as well as RNA levels in GA-treated cells compared to vehicle-treated cells. We also examined the distribution of EV71 at 1 hour post infection in GA-treated RD cells using immunofluorescence of confocal microscope. Cellular EV71 spreaded in the whole cells with 0 µm GA (no treatment) compared to very few EV71 distributed in the cytosol and submembrane of cells treated with 2 µM GA, and even most retained in the submembrane while cells received 10-fold more of GA (Figure 4C). EV71 colocalized with HSP90 in the submembrane of infected RD cells in the early infection was confirmed (Figure 4D). These results demonstrate that HSP90 does associate with EV71 and supports EV71 entry.

**Figure 5:**
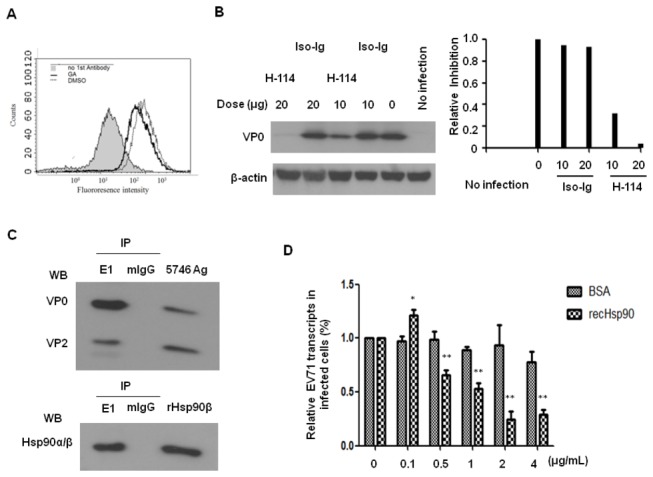
HSP90 interacts with EV71 on the surface of infected cells. (A) RD cells were treated with 2 µM (gray curve) or 0 µM (0.1% DMSO; black curve) of GA for one hour and then washed with 1xPBS before incubated with anti-HSP90 antibody (N-17) or control goat IgG (filled curve) at 4°C for another one hour. The cells were washed and fixed by 2% paraformaldehyde in PBS for 30 min. After washing, the cells were stained with anti-goat IgG-TR antibody and analyzed by the FACScan flow cytometer. (B) Pretreatment of RD cells with 10 and 20 µg of anti-HSP90 antibody (H-114) or control rabbit IgG (Iso-Ig) at 4°C for one hour before MOI=0.1 of EV71 5746 infection was conducted. After one hour infection, cells were washed with medium and incubated for another 16 hours. Viral capsid proteins in the cells were detected by western blot using MAB979 antibody. The internal β-actin was detected by blotting the same membrane with monoclonal anti-β-actin antibody. Corresponding changes in EV71 VP0 levels compared to the expression levels in the cells without antibody treatment as 1.0 were quantified using Image-Pro Plus 6.0 software. (C) Performance of co-immunoprecipitation of EV71 5746 and recombinant HSP90β by anti-EV71 E1 antibody or non-immune mouse IgG (mIgG) following by immunoprecipitation with protein G-agarose was described in the Materials and Methods. Precipitates were washed and subjected to detect the associated EV71 capsid proteins and HSP90 by blotting with MAb979 and anti-HSP90 antibodies, respectively. (D) MOI =0.1 of EV71 5746 was pre-mixed with various amounts of recombinant HSP90β or bovine serum albumin (BSA) and incubated at 4°C for overnight. EV71-HSP90β or EV71-BSA mixtures were inoculated into 12-well plate of RD cells and incubated on ice for 90 min. Followed by washing the unbound EV71 away, total RNA were extracted to quantify EV71 transcripts as described in the legend of Figure 4. Each normalized 2^Ct^ value from EV71-HSP90β mixtures-treated cells was ratio to the value from the value of 2^Ct^ obtained from the only EV71-treated cells that represented as 100%. A schematic representation of the percentage of EV71 attachment was shown. Unpaired student *t* test with Welch correction was used for statistical analysis.

To confirm that the inhibition of virus level by GA was not due to the down regulation of cell surface HSP90, flow cytometric analysis of HSP90 expressed on the surface of RD cells pre-treated with GA or vehicle was carried out. It was seen that HSP90 expression on RD cell surface was not affected by GA treatment (Figure 5A). This result has been confirmed by previous study [40,41]. GA disrupted the stabilizing interaction of Hsp90 with ErbB2/HER2 [42] that resulting in the degradation of ErbB2/HER2 was reported [43,44], To prove the activity of HSP90 was impaired by GA treatment, we examined the expression of HER2 in RD cells which pre-treated with the same dose of GA. Flow cytometry showed that HER2 was downregulated in GA-treated cells compared to vehicle-treated cells (Figure S2), indirectly confirming the function of HSP90 was impaired by GA. These results together demonstrate that HSP90 plays a role in EV71 entry of RD cells.

### HSP90 directly interacts with EV71

We further studied whether HSP90 facilitates EV71 entry by direct binding to EV71 virions on the cell surface. The ability of HSP90 specific antibody to inhibit EV71 infection of RD cells was analyzed. RD cells were pre-treated with various amounts of anti-HSP90 antibody (H-114) or isotype rabbit Ig before EV71 5746 infection and the expression of viral capsid proteins was detected by Western blot. Inhibition of EV71 infection by H-114 antibody was in a dose-dependent manner; 20 µg of H-114 antibody completely inhibited EV71 entry and therefore VP0 production, while 10 µg antibodies only partially inhibited VP0 production compared to the same amount of control IgG (Figure 5B).

To examine whether HSP90 physically interacts with EV71 in the cells, co-immunoprecipitation of EV71 virions with recombinant HSP90β (rHSP90β) was carried out. A monoclonal E1 antibody (generated by us) only recognizes the adequate stereo structure of EV71 particle [45]. Western blotting of the immunoprecipitates with antibodies against VP0 and VP2 demonstrated that EV71 virions were indeed co-precipitated from E1 antibody-protein G mixture but not from the non-immune mouse IgG (mIgG)-protein G mixture. Direct immunoblotting of the same amount of EV71 particles as antigen that corresponding the molecule size of EV71 capsid proteins were observed (the upper panel of Figure 5C). HSP90 was also detected in co-immunoprecitates from EV71-rHSP90β pulled down by E1 antibody-protein G agarose but not by control Ig-protein G agarose, referenced to the Western blotting of rHSP90β antigen, confirming that HSP90 directly interacts with EV71 (the bottom panel of Figure 5C).

Competition of EV71 binding to HSP90 was carried out using recombinant HSP90β. EV71 5746 virions were pre-mixed with various amounts of recombinant HSP90β and then inoculated into RD cells. EV71 RNA transcripts in the cells were quantified by real-time RT-PCR. The results showed that recombinant HSP90β competed with EV71 entry in a dose-dependent manner (Figure 5D). These results demonstrate that EV71 physically interacts with HSP90 while EV71 infection.

### HSP90 inhibitor cannot influence the transcription levels of EV71 capsid proteins

**Figure 6:**
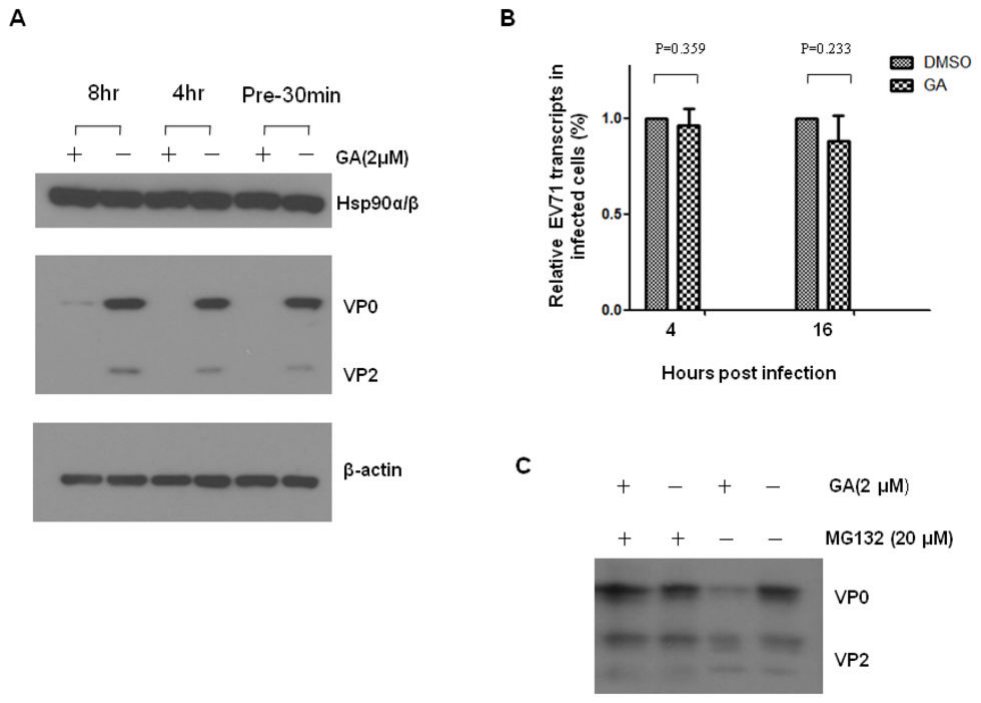
GA does not inhibit EV71 RNA transcription but accelerates the proteosomal degradation of *de novo* synthesized capsid proteins. RD cells were treated with 2 µM of GA by 30 min early of MOI= 0.01 of EV71 5746 infection and incubated for 16 hours in the presence of drug. Alternatively, cells were treated with GA at 4 or 8 hours post infection and then continued the incubation up to 16 hours. (A) Cell extracts were prepared to detect EV71 capsid or HSP90 proteins by immunoblotting with Mab979 antibody or anti-HSP90 antibody (N-17), respectively. Cellular β-actin was detected by blotting the same membrane with monoclonal anti-β-actin antibody. Data represent one of two independent experiments. (B) RNA were prepared at 4 and 16 hours post infection to quantify EV71 transcripts as described in the legend of Figure 4. Each β-actin normalized 2^Ct^ value from GA-treated cells was ratio to the value from the value of 2^Ct^ obtained from the vehicle-treated cells. (C) RD cells were treated with vehicle, or 2 µM of GA, or 20 µM of MG132 alone, or GA plus MG132, at 4 hours post EV71 infection and continued the incubation for 12 hours. The extracts were prepared to detect viral capsid proteins by Western blotting using Mab979 antibody. Data represent one of two independent experiments.

HSP90 is known to facilitate folding of nascent proteins that participate in cell metabolism and function [46]. The role of HSP90 in EV71 life cycle in infected cells was further studied by inhibiting HSP90 using GA. RD cells were treated with either GA 30 min before or 4 or 8 hours after EV71 5746 inoculation. EV71 infection was inhibited by all the above treatments with GA (Figure 6A). This inhibition by GA was not due to down-regulation of cellular HSP90 (the upper panel of Figure 6A).

To investigate whether GA inhibits EV71 replication, the level of EV71 RNA transcripts was examined in the same experiment. Four hours after EV71 infection, RD cells were treated with GA or vehicle, and RNA transcripts targeted by VP1 specific primers were detected by real-time RT-PCR at 4 and 16 hours after GA treatment. No inhibition of EV71 transcripts expression was seen in GA-treated cells compared to vehicle-treated cells (Figure 6B). We further addressed the question, whether the inhibition of EV71 production by GA treatment was due to increase in the proteosomal degradation of newly synthesized EV71 capsid proteins. We used MG132 (carbobenzoxy-Leu-Leu-Leucinal), a peptide aldehyde, which effectively blocks the proteolytic activity of the 26S proteosome complex [47]. To this end, RD cells were added with GA in the presence or absence of MG132 at 4 hours post EV71 infection and then cultured for another 12 hours, EV71 translated proteins in the cells were analyzed by western blot. Viral capsid proteins were thin in EV71-infected cells treated with GA in the absence of MG132 compared to the infected cells without GA treatment (Figure 6C). In contrast, synthesized capsid proteins were increased in the infected cells treated with GA in the presence of MG132. RD cells treated with MG132 alone did not affect EV71 capsid proteins expression (Figure 6C). These results confirm that HSP90 plays a role in protecting newly synthesized EV71 capsid proteins from proteosomal degradation during viral replication.

### HSP90 participates in the assembly of EV71 viral particles

**Figure 7:**
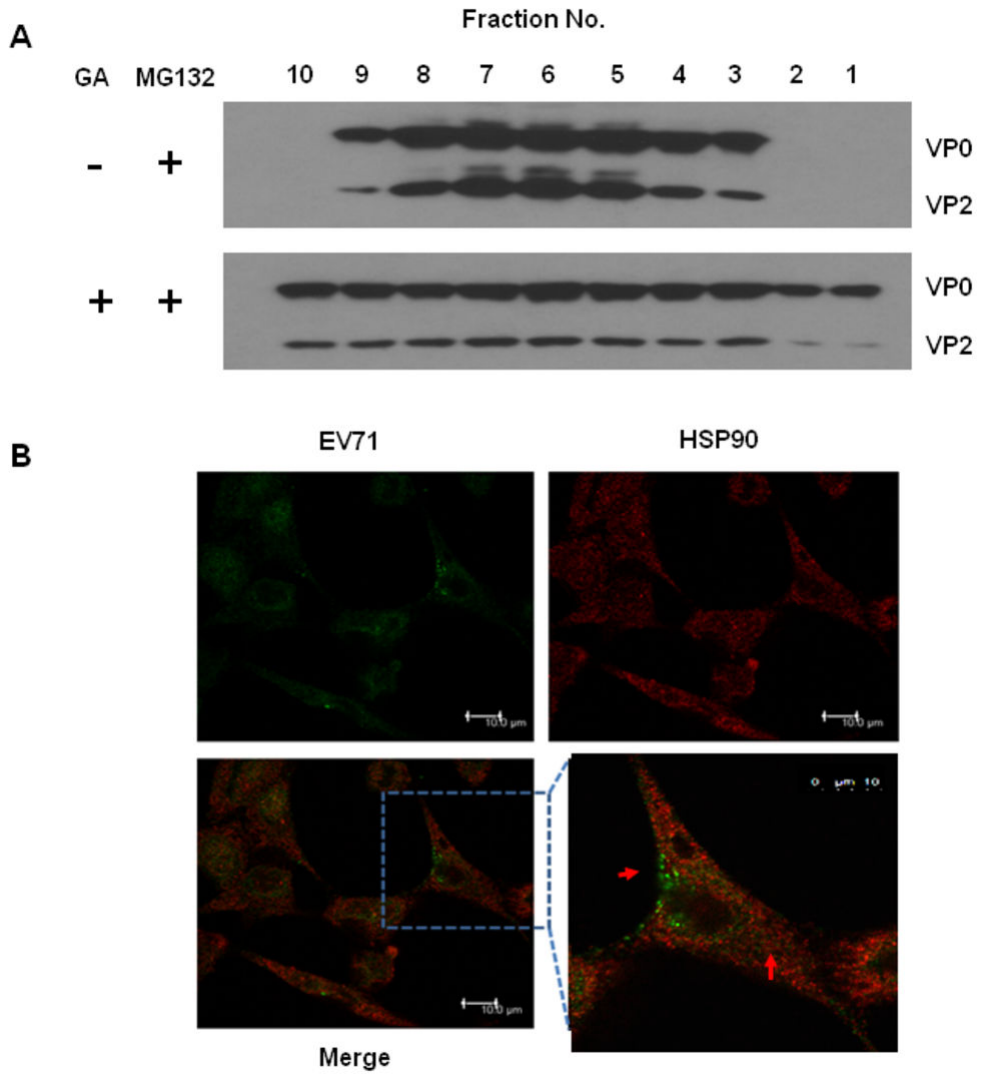
HSP90 colocalizes and participates in the assembly of EV71. (A) RD cells were treated with vehicle or 2 µM of GA in the presence of MG132 and followed by MOI=0.1 of EV71 5746 infection was performed. Cell lysates were prepared at 16 hours post infection and fractionated in iodixanol density-gradients as described in the Materials and Methods. Each fraction was subjected to Western blot using Mab 979 antibody. (B) RD cells grew on coverslips prior to the infection of EV71 5746 (MOI = 5). After infection, cells were washed with medium and incubated at 37°C for 12 hours before fixed with cold methanol. HSP90 and EV71 were detected by adding of anti-HSP90 (H-114) and MAb979 antibodies and stained with the respective secondary antibodies conjugated with fluorescence dye as described in the Materials and Methods. Detection of cellular HSP90 (red), EV71 (green), and EV71-HSP90 merging (yellow) using a confocal microscope Leica TCS SP5 II was performed. The pictures were taken at 200x or 630x magnification from 2 independent experiments and one of them was shown.

To annotate the role of HSP90 in EV71 replication, synthesized viral particles in HSP90 inhibitor-treated cells were fractionated in iodixanol density-gradients, since GA treatment facilitates proteosomal degradation of the capsid proteins (Figure 6C). RD cells were pretreated with 2 µM of GA in the presence of MG132 followed by infection with EV71 5746MOI=0.1. Cell lysates were centrifugally fractionated on 16 hours post infection and EV71 capsid proteins in each fraction were analyzed by Western blot. Without GA treatment, nascent EV71 cased proteins were located restrictedly in the lysate fractions 3-9 that contained assembled viral capsids (Figure 7A). However, with GA treatment, nascent capsid proteins including mature and immature forms were widely distributed in all the fractions (Figure 7A). We also visualized the location of HSP90 and EV71 in RD cells in confocal microscope. EV71 (green) and HSP90 (red) distributed in the submembrane and cytosol of cells were found (Figure 7B). After merging, HSP90 and EV71 capsid proteins (yellow) were colocalized in the cytosol and some in the submembrane site, and interestingly, some newly formed viral particles in the cytosol was dissociated with HSP90 (green) (Figure 7B). These results confirm the physical interaction of HSP90 and EV71 (Figure 5C) and HSP90 participates in the assembly of EV71 capsid in the late stage of virus production. This is also supported by the previous study that HSP90 binds newly synthesized P1 translates of polio virus and protects them from proteosomal degradation to form the mature capsid proteins leading to viral assembly [35].

### Targeting of HSP90 by 17-AAG conferred protection against EV71 infection in hSCARB2-transgenic mice

**Figure 8:**
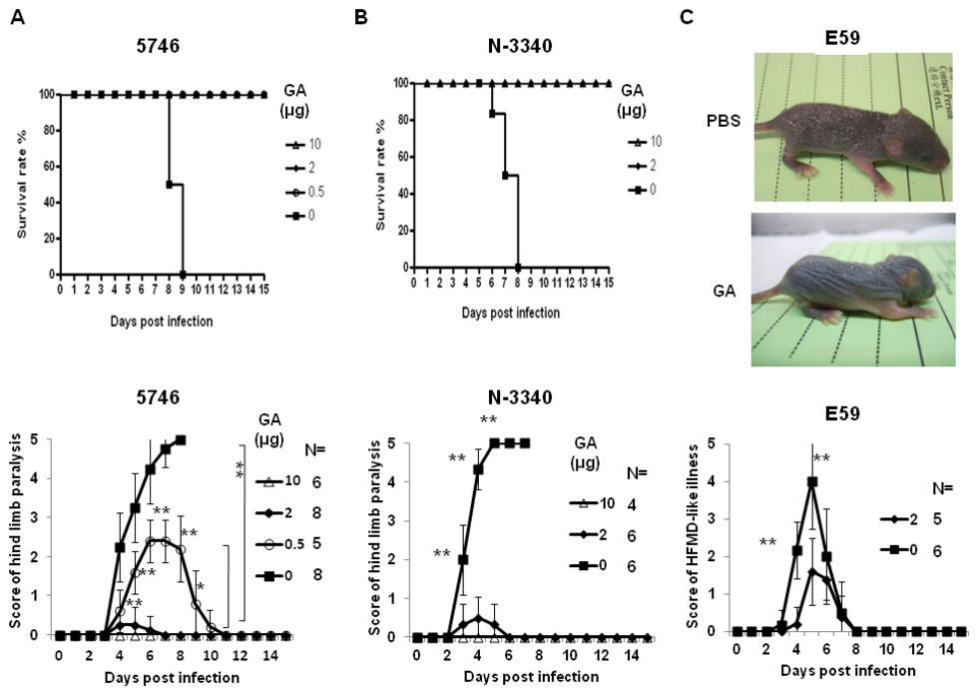
17-AAG confers protection against the different genotypes of EV71 in hSCARB2-transgenic mice. Seven-day old mice subcutaneously preinfected with 3x10^6^ pfu of (A) EV71 5746 (C2) or (B) N-3340 (C4), or (C) one-day old mice preinfected with 1x10^7^ pfu of EV71 E59 (B4), were given various dose of 17-AAG intraperitoneally twice at the time points of 4 hours and 24 hours after infection. The same age of infected mice treated with the same volume of vehicle (10% DMSO plus 5% glucose) as control were included. Mice were monitored daily and the survival rates and the score of HLP were recorded in (A) (B), and the score of HLS was recorded in (C). The criterion to score the severity of HLP and HLS was described in the Materials and Methods. The mean of score from each group was calculated and the number of mice (N) in each group was shown. 100x magnification of pictures (C) shown the representative data were taken on day 5 post infection. The Logrank test for survival rate and One-way ANOVA with the Kruskal-Wallis test for the score of HLP and HLS were calculated for statistical analysis.

We further questioned whether targeting of HSP90 by inhibitors could protect animals from the lethal challenge of EV71 *in vivo*. Human SCARB2-transgenic mice had been generated by us and proven that they can serve as a reliable infectious model, with the development of severe neurological diseases leading to death upon challenge with EV71 5746-TW98 (C2) or N-3340 (C4) or development of severe HFMD associated with recoverable neurological diseases by E59 (B4) or N2838 (B5) strains [22]. Geldanamycin is known as a potent antitumor agent [48]; however, it has not been used in clinical trials because of its liver toxicity [49]. *17-AAG* is a new derivative of geldanamycin that shares its important biological activities [50] but shows less liver toxicity [51]. 7-day old hSCARB2-transgenic mice were preinfected with 3x10^6^ pfu of EV71 5746 (Figure 8A) or N-3340 (Figure 8B) strain subcutaneously followed by administration of different dose of 17-AAG or vehicle intraperitoneally on 4 and 24 hours post infection. Mice were monitored for survival and hind limb paralysis (HLP) on a daily basis. For vehicle-treated transgenic mice, progressive HLP peaked on day 8 (5746) or day 5 (N-3340) post infection and then the animals were completely died on 8-9 days after infection. 5746-infected mice that received as low as 0.5 µg of 17-AAG were protected from the development of severe HLP and 100% of animals were rescued. Administration of 2 µg 17-AAG completely inhibited HLP syndrome and all mice survived (Figure 8A). Mice developed a lethal HLP by N-3340 challenge were fully protected by the injection of minimal dose (2 µg) of 17-AAG (Figure 8B). Moreover, mice received no drugs developed severe HFMD-like hair loss associated with scurf (HLS) which peaked on day 5 and then recovered on day 8 post infection while challenged with 1x10^7^ pfu of E59 (Figure 8C). However, treatment of 2 µg 17-AAG significantly inhibited HLS formation (Figure 8C). Taken together, our result suggests that targeting of HSP90 by 17-AAG can be a new therapy to control EV71 infection.

## Discussion

Viruses must deliver their nucleic acids across a cellular membrane into the cytoplasm of their target cell to cause infection [52,53]. The strategy employed by EV71 is the use of their capsid proteins especially VP1, which recognizes a receptor on the cell surface (like Human SCARB2 [21]; or PSGL-1 by EV71 [20]); following binding, the virion is internalized to an endosome. A reduced pH in the endosome causes conformational changes in the capsid proteins, resulting in fusion of the viral membrane with the endocytic vesicle membrane and subsequent release of viral RNA into the cytosol for replication. The cellular receptors or adaptors that mediate the attachment and entry of EV71 have been characterized [20,21,24,54] but there remained some uncertainties. Based on our report that EV71-HSP90 interaction was shown to be common to EV71 clinical isolates of selected genotypes-C2, C4, and B4, and others- that have not been proved yet, suggesting that interaction with HSP90 could be a common feature of circulating EV71 strains. Some studies have revealed that HSP90β plays as a cellular factor for Japanese encephalitis virus [55] infection and Epstein-Barr virus replication [56], or isoform-unidentified HSP90 plays as components of receptor complex for dengue virus [57] or for infectious bursal disease virus [58] infection. In our study, HSP90β instead of HSP90α participating in EV71 infection was also observed. Indeed, we examined whether the interaction of HSP90 with other reported receptor or co-receptor like SCARB2 [21] or Annexin II [24] in EV71-infected cells. We did not find any interaction using co-immunoprecipitation (data not shown). In addition, mouse HSP90 shares high homology to human HSP90 in terms of amino acid sequence but it cannot support EV71 infection into mouse cells [54]. Overexpression of human HSP90α or HSP90β, or both HSP90α and HSP90β in mouse fibroblast NIH3T3 cells cannot support EV71 infection (data not shown). Therefore, human HSP90 might function as a cofactor for EV71 entry. When the binding of EV71 and its receptor occurs, HSP90 might interact with EV71 particles on the cell surface thus triggering virus internalization. Alternatively, EV71 might first attach to HSP90 and then facilitate EV71 accumulated on the surface to associate with its receptor for cellular entry. Therefore, how physical association of HSP90 with EV71 capsid proteins occurs coordinate with EV71 receptor(s) are areas that can only be addressed through conducted another set of studies.

HSP90 also plays a role as a chaperone for interaction with a wide variety of cellular proteins, and supports protein function by folding to form an adequate structure [32,33]. Here we found that HSP90 did not affect viral RNA transcription but facilitated the stability of synthesized viral capsid proteins in the cells by inhibition of post-translational proteosomal degradation that helps EV71 virion assembly. This information indicates the multiple roles of HSP90 in supporting EV71 infection as well as a chaperone function.

Our study is the first to demonstrate that HSP90 can be a target to control EV71 infection. GA and its synthetic derivatives show higher affinity to HSP90 in tumor cells as compared to normal tissues and constitute a class of potential antitumor drugs [31,39]. GA only inhibits the function of HSP90 involved in EV71 entry and assembly but has no anti-viral activity on EV71 because the expression of viral transcripts in the infected RD cells pretreated with GA was not affected (Fig. 6B). However, the study is not able to exclude the possibility that HSP90 may not be involved in virus entry and assembly but inhibited HSP90-dependent cellular functions that can indirectly influence viral replication. Using 17-AAG, a gleldanamycin analog which is less toxic and recognized to be an anti-tumor [51] and anti-ameliorates polyglutanime-mediated motor degeneration agent [59], effectively protected hSCARB2-Tg mice from the challenge with C2, C4, and B4 genotypes of EV71. Coxsackievirus (CV) is also a causative agent for HFMD beside of EV71 and has been proved that it is virulent in hSCARB2-mice [22]. However, treatment of 17-AAG might not protect hSCARB2-mice from the lethal challenge of CVA16 strain (Figure S3). These results indicate the role of HSP90 in the infection of CV may be different from EV71. Therefore, the successful application of 17-AAG to enterovirus infection remains to be seen especially with the other genotypes of EV71 and CV isolates.

**Figure 9:**
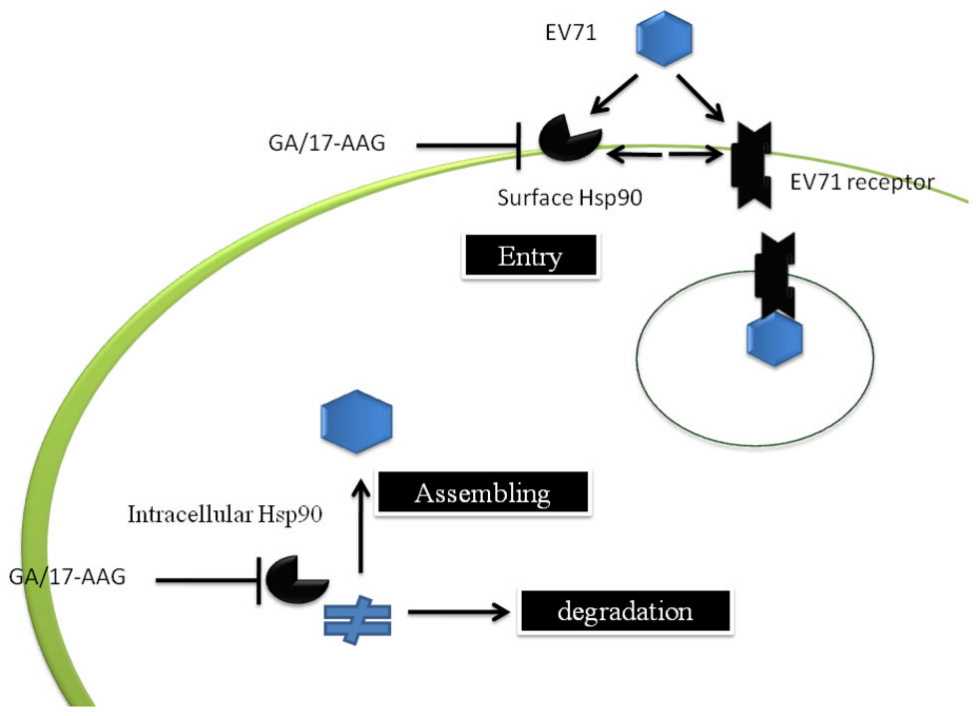
Model illustrating how HSP90 interacted with EV71 and supported EV71 infection. In the infection, EV71 particles attach to the surface receptors (such as human SCARB2 or PSGL-1), HSP90 expressed on the surface may following bind to EV71 and help for virus entry. The newly synthesized viral capsid proteins may bind to HSP90 to avoid being degraded by proteosome that facilitates viral assembling. Targeting of HSP90 using the inhibitor geldanamycin (GA) and its analog, 17-allyamino-17-demethoxygeldanamycin (17-AAG), can efficiently control EV71 infection based on the blockage the described pathways that HSP90 involved.

In conclusion, we have demonstrated that HSP90β, but not HSP90α, is involved in EV71 entry by direct binding of EV71 to HSP90β on the cell surface. After viral entry, HSP90β protects viral proteins from proteosomal degradation and helps viral assembly. The similar observations also has been reported recently [60]. A model depicting these pathways was shown in Figure 9. Targeting of HSP90 by its inhibitors, especially 17-AAG, demonstrated its efficacy as an anti-EV71 therapeutic agent in an EV71 infectious mice model. Our study has considerably extended the therapeutic application of 17-AAG beyond oncological and neurogenerative diseases. HSP90 inhibitors have the potential for widespread application towards the control of EV71.

## Materials and Methods

### Ethics statement

All animal experiments were conducted in accordance with the guidelines of the Laboratory Animal Center of the National Health Research Institutes (NHRI), Taiwan. The animal-use protocols were reviewed and approved by the NHRI Institutional Animal Care and Use Committee (Approved protocol no. NHRI-IACUC-101006-A). EV71 5746-TW98 (C2) and N-3340 (C4) strains elicit a lethal neurological virulence of hind limb paralysis (HLP) and E59 (B4) induces HFMD-like hair loss associated with scurf (HLS) and a non-lethal HLP in hSCARB2-transgenic mice [22]. In the protection of genotype C of EV71 challenge, survival rate was used as one of end points to assess the protective efficacy of anti-EV71 medication. Survival rate used as an index of pathogenesis of EV71 infection in experimental animal models has been reported by many studies [15,17,18,22]. In B4 challenge test, scoring of mice with HLS was performed. After the investigation, tested animals were euthanasia by 100% CO_2_ inhalation for 5 minutes (min) followed by cervical dislocation to minimize the animals suffering. To perform virus challenge, mice are explored in the anesthetic inhalator chamber containing isoflurane (For initial phase: 5%, for maintainced phase: 1.5%~2.5%) for 1 min before subcutaneously or intraperitoneally immunized with EV71 or drugs, respectively.

### Cells, viruses, compounds, and antibodies

African green monkey kidney (Vero) (ATCC No. CCL-81) and human rhabdomyosarcoma (RD) (ATCC No. CCL-136) cells were provided by the Taiwan Centers of Disease Control (Taiwan CDC); the original cell lines were obtained from the American Type Culture Collection (ATCC), USA. Vero cells were cultured in a VP-SFM medium (Gibco-Invitrogen, CA, USA) supplemented with 4 mM L-glutamine (Gibco-Invitrogen, CA, USA) and the RD cell line was cultured in a DMEM medium with 10% fetal bovine serum (Gibco-Invitrogen, CA, USA). They were maintained in a 37°C incubator equilibrated with 5% CO_2_. Two clinically isolated strains of EV71, E59 (B4) (GenBank: GQ150746.1) and Tainan/5746/98 (C2) (GenBank: AF304457.1) were obtained from Dr. Jen-Ren Wang, National Chen-Kung University, Tainan, Taiwan and were propagated in Vero cells based on the microcarrier cell culture bioprocess previously reported [61,62]. The virus stocks were stored at -80°C. The titer of virus stocks was tested in a standard plaque-forming assay as described previously [54]. The number of plaque-forming units (pfu) was calculated.

Geldanamycin (GA; Cat. No. ant-gl) and 17-allyamino-17-demethoxygeldanamycin (17-AAG; Cat. No. ant-agl) were purchased from InvivoGen, CA, USA MG132 (Cat. No. c2211) was purchased from Sigma-Aldrich, MO, USA. Recombinant HSP90β (Cat. No. SPR-102) was obtained from Stress Marq Biosciences Inc., British Columbia, Canada.

Monoclonal antibody, Mab979 recognized VP0/VP2 capsid protein of EV71 [45] was purchased from Millipore, Inc., MA, USA A VP1-specific monoclonal antibody E1 produced in house had been described [45]. Two anti-HSP90α/β antibodies, N-17 (Cat. No. sc-1055) and H-114 (Cat. No. sc-7947), and donkey anti-goat IgG-TR antibody (Cat. No. sc-2783), normal rabbit IgG (Cat. No. sc-2027), and normal goat IgG (Cat. No. sc-2028) were purchased from were purchased from Santa Cruz Biotechnology, Inc, CA, USA. Specific antibodies to HSP90α (Cat. No. 07-2174) or HSP90β (Cat. No. AB3468) were purchased from Millipore, Inc., MA, USA. Antibodies specific to human β-actin (Cat. No. A5441) was purchased from Sigma-Aldrich, MO, USA. Horse radish peroxidase (HRP)-conjugated donkey anti-mouse antibody (Cat. No. 715-036-150) or HRP-conjugated rabbit anti-goat antibody (Cat. No. 305-035-003) were purchased from Jackson Immunoresearch, Inc., PA, USA.

### Down-regulation of cellular gene expression by siRNA

RD cells at 80% confluence were transfected with siRNA by liposome-oligonucleotide transfection method. Specific siRNA to HSP90α/β gene (Cat. No. 202611C05, Invitrogen, CA, USA) with the control siRNA (Invitrogen, CA, USA), or siRNA specific to HSP90α gene (three oligonucleotides mixture, Cat. No. sc-29353A/B/C, Santa Cruz Biotechnology, Inc, CA, USA) or HSP90β gene (three oligonucleotides mixture, Cat. No. sc-35606A/B/C, Santa Cruz Biotechnology, Inc, CA, USA) with the control siRNA (Santa Cruz Biotechnology, Inc, CA, USA) were used. Briefly, 250 µL Opti-MEM (Invitrogen, CA, USA) containing 200 pmoles of siRNA pre-mixed with 12 µL of TurboFect siRNA transfection Reagent (Fermentas, MD, USA) was added per well of a 6-well plate and incubated at 37°C for 24 hours in an incubator. After incubation, the cells were infected with EV71 (MOI = 0.01) in serum-free culture medium followed by 1 hour incubation at 37°C before washing three times with serum-free culture medium. After 16 hours of incubation, cells lysates were prepared for western blot.

### Western blot

The Western blot was performed according to previous report [54]. Total cell lysates were prepared by treatment of 1-2 x 10^6^ cells in 100 µl of ice-cold lysis buffer (0.5% sodium deoxycholate, 0.1% sodium dodecyl sulfate (SDS), 0.5% NP-40, 50 mM TRIS, 150 mM NaCl) with the addition of protease inhibitors cocktail (Roche, French) and 1 mM PMSF (Sigma-Aldrich, CA, USA). Lysates were then centrifuged for 20 min at 10,000 rpm, at 4°C to sediment the cell debris. To prepare cytosolic fraction, we followed the standard protocol described in the Chapter 9, the sixth edition of Molecular Cell Biology (edited by Harvey Lodish, the publisher; W.H. Freeman and Company, New York, USA). Briefly, the cells were homogenized in subcellular fraction buffer (250 mM sucrose, 20 mM HEPES pH=7.4,10 mM KCl, 1.5 mM MgCl_2_, 1 mM EDTA, 1 mM DTT) with the addition of protease inhibitors cocktail. Cell debris and nuclei were pulled down by 100xg, 5 min and then 15,000xg, 5 min centrifugation. Cytosolic fraction and membrane fraction were separated by centrifuge 300,000xg for 2 hours. The protein concentration of the cell lysates or fractions was measured by Bradford method [63]. Cell lysate containing 10 µg of proteins was mixed with loading dye and loaded per well of a 10% SDS-polyacrylamide gel (SDS-PAGE, Amersham Biosciences-GE Healthcare, USA) and subjected to electrophoresis in 1X Tris-glycine SDS-running buffer. The resolved proteins were transferred onto a nitrocellulose membrane (Hybond-ECL, Amersham Biosciences-GE Healthcare, USA). Protein-containing membrane was soaked in 5% skim milk in 1x PBS for 30 min at room temperature, then washed three times with 1x PBS with 0.05% Tween 20 (PBS-T). The membrane was incubated with MAB979 antibody (1:5000), or anti-Hsp90α/β antibody (N-17; 1:1000), or specific antibodies to HSP90α (1:1000) or HSP90β (1:1000) for 14-16 hours at 4°C, and subsequently washed with PBS-T followed by incubation with HRP-conjugated donkey anti-mouse secondary antibody (for MAB979, Hsp90α, or Hsp90β) or HRP-conjugated anti-goat secondary antibody (for N-17). After 1 hour incubation, the membrane was washed 5 times with PBS-T, and then Super Signal West Pico chemiluminescent substrate (Pierce, IL, USA) was layered onto the membrane and then the membrane was exposed to an X-ray film (Kodak, NY, USA). When necessary, the membranes were stripped using Restore buffer (Pierce, IL, USA) and blotted with another antibody. The blots were quantified using Imag–Pro Plus 6.0 software.

### Determination of virus titers in siRNA-treated cells

The culture mediums from EV71-infected RD cells pre-treated with siRNA were harvested at 12, 24, 36, and 48 hours post virus infection and centrifuged at 1000xg for 5 min. The virus titers in the supernatant were determined using the median endpoint of the tissue culture’s infectious dose (TCID_50_) as described previously by Liu et al. [45]. The TCID_50_ values were calculated using the Reed–Muench method [64].

### Treatment of EV71-infected cell with inhibitors

5x10^5^ RD cells seeded in a 6-well plate one day prior to the experiments were treated with various concentrations of the inhibitors for one hour and then infected with EV71 (MOI = 0.01) in serum-free culture medium for 1 hour at 37°C in the presence of the inhibitor. The cells were washed three times with serum-containing culture medium and additionally cultured for 16 hours. Alternatively, RD cells were infected with EV71 before inhibitor treatment. After 1 hour infection, the infected cells were washed and treated with inhibitors for different times, washed and then cultured for 16 hours. The cells were harvested to prepare RNA or cell lysate for real-time RT-PCR or western blot, respectively.

### Flow cytometry

RD cells were harvested using trypsin-free dissociation buffer (Gibco, Invitrogen, CA, USA) and spun down to remove the supernatant. Cells were washed once with 1x PBS (pH = 7.4) and then incubated with anti-HSP90α/β antibody, N-17 (1:100) or control IgG at 4°C for 1 hour. Cells were washed three times with 1x PBS and fixed with 2% paraformalydehyde in PBS at room temperature for 30 min. The cells were washed again and 1 mL of donkey anti-goat IgG-TR secondary antibody (1:400) was added and incubated at 4°C for 1 hour. After the incubation, the cells were washed and HSP90-expressing cells were detected using FACScan flow cytometer and analyzed using the CellQuest software (Becton Dickinson Immunocytometry System).

### Real-time RT-PCR

Total RNA was purified from RD cells using the TRIZOL reagent (Invitrogen, CA, USA) following the manufacturer instructions and subjected to real-time RT-PCR. Total RNA were converted into cDNA using random primers (Genomics BioSci&Tech, Taiwan) and reverse transcriptase (Bionovas, Toronto, Canada). The synthesized cDNA was subjected to quantitative PCR analysis (the LightCycler® 480 SYBR Green Real-Time PCR system) using the primer pairs specific to the target genes. Human β-actin gene expression was used as internal control. The conditions used for PCR were: 95°C for 3 min; followed by 40 cycles of 95°C for 10 second (s), 65°C for 20 s, and 72°C for 2 s; followed by incubation at 72°C for 2 min. The number of cycles required for amplification of the target gene was obtained. The relative expression of the target gene was calculated as follows: the individual Ct obtained from the experimental group or control group was subtracted by its respective Ct (β-actin), and then 2^Normalized Ct (target gene from the sample without drug treatment)^ was divided by 2^Normaliszed Ct (target gene from the sample with drug treatment)^. Primer pairs targeting to VP1 region of EV71 E59 and 5746-TW98 RNA transcripts and human β-actin were listed in Table 1. All primer sets were synthesized commercially by Genomics BioSci&Tech, Taiwan.

**Table 1:** List of the sequence of primer pairs specific to target genes.

**Target gene**	**5’ to 3’ sequence of oligonucleotide**
**5746 VP1**	F: ACGCGCAAATGCGTAGAAAGGT
	R: TTAGTGGCAGTTTGCCATGCGA
**E59 VP1**	F: AGAGAGTCACTTGCTTGGCAGACA
	R: ACGACTAGTGCCGGTCGGTTTAAT
**β-Actin**	F: ACCAACTGGGACGACATGGAGAAA
	R: TAGCACAGCCTGGATAGCAACGTA

F: forward primer. R: reversed primer

### Immunofluorescence

Immunofluorescence assays were conducted on RD cells grew on coverslips after EV71 (MOI= 5) incubation at 37°C for 1 hour and then washed with medium and cultured at 37°C for another 12 hour. After wash by PBS, cells were fixed with cold methanol and then incubated with a mixture of primary antibodies; anti-EV71 antibody (Mab979; 1:400) accompanied by anti-HSP90 (N-17; 1:400). Then fluorescence signals were conducted by hybridization of secondary antibodies; fluorescein isothiocynate (FITC)-conjugated anti-mouse IgG antibody (1:400; BioLegend) accompanied with TR dye-conjugated anti-rabbit IgG antibody (1:400; sc-2780, Santa Cruz Biotechnology, Inc, CA, USA).

For staining of EV71 in GA-treated or untreated cells, RD cells pre-treated with various dose of GA or 0 µM GA (0.1% DMSO) as control for 1 hour before EV71 infection. After 1-hour infection, cells were fixed with cold methanol. Cells were incubated with Mab979 antibody (1:1000) alone or Mab979 (1:1000) and anti-HSP90 (N-17; 1:400) antibodies, and then stained with FITC-conjugated anti-mouse IgG antibody or accompanied with TR dye-conjugated anti-rabbit IgG antibody. Nuclei were stained by 4',6-diamidino-2-phenylindole (DAPI). Signals were detected using a Leica TCS SP5 II confocal microscope. The thickness of the slide sectionedly scanned by lasering of fluorescence confocal microscopy was 0.6 ~1 µm.

### Co-Immunoprecipitation

10^4^ pfu of EV71 were mixed with 1 µg of recombinant HSP90β (Cat. No. SPR-102, StressMarq Biosciences Inc., Canada) and incubated at 4°C for 5 hours with occasionally rocking. Following HSP90β-viruses mixtures was rocked with 5 µg of anti-EV71 E1 antibody or non-immune mouse IgG at 4°C for overnight prior to be immunoprecipitated by adding protein G agarose beads (Santa Cruz Biotechnology) for another 3 hours. Precipitates were washed 5 times with Opti-MEM medium and then resuspended in 50 µL SDS loading buffer, boiled for 5 min and then 20 µL of samples were taken and subjected to SDS-PAGE and western blotting with MAB 979 and anti-HSP90 antibodies individually. 10^4^ pfu of EV71 and 1 µg of recombinant HSP90β directly subjected to immunoblot as positive controls were included. Detection was achieved by using appropriate secondary antibodies labeled with HRP as described above. Each experiment has been repeated at least two times independently.

### Density gradient separation of cytosolic fractions

5 x 10^5^ RD cells seeded in a 6-well plate one day prior to the experiments were infected with MOI = 0.01 of EV71 5746 before the inhibitor treatment. After 1 hour of infection, the infected cells were washed and incubated for 4 hours before treated with 2 µM of GA or vehicle in the presence of 20 µM MG132 for 12 hours. The cells were harvested and lysed in 0.5% NP-40 lysis buffer (50 mM Tris/HCl, pH 7.5, 150 mM NaCl, 2 mM EDTA, and 0.5% NP-40) on ice for 30 min and then centrifuged at 12000 rpm for 5 min to remove the cell debris. The supernatants were harvested. Self-generated iodixanol gradients were prepared by mixing 0.25 mL supernatants, 330 mL solution S (0.25 M sucrose, 15 mL EDTA, 30 mM Tris/HCl, pH 8.0), and 0.42 mL 60% (w/v) iodixanol (Cat. No. 1114542, Optiprep; Axis Shield, UK) to form a homogenous solution. Gradients were generated by centrifugation at 162000xg for 24 hours at 4°C. The different fractions were harvested manually from the top (named as fraction No. 1), 0.1 mL per fraction and serially 10 fractions were collected for each sample. These fractions were subjected to Western blot using Mab979 antibody.

### Therapy of 17-AAG in hSCARB2-transgenic mice challenged with EV71

Human SCARB2 (hSCARB2)-transgenic mice in C57BL/6 background generated by our group were maintained by cross-mating hSCARB2-transgenic individuals to obtain inbred mice [22]. 7-d-old hSCARB2-transgenic mice were inoculated subcutaneously with 3x10^6^ pfu of genotype C of EV71 including 5746 (C2) and N-3340 (C4) strains, or PBS alone. 1-d-old hSCARB2-transgenic mice were inoculated subcutaneously. with 1x10^7^ pfu of genotype B of EV71, E59 (B4) strain. For protection studies, 17-AAG were injected intraperitoneally into the mice after 4 and 24 hours after infection. The mice were monitored daily for survival. The severity of HFMD-like and paralysis symptoms was scored from 0 to 5 using the following criteria [22]: For HFMD-like symptoms 5=80% hair loss associated with scurf (white spots) (HLS), 4=50% HLS, 3= >30% HLS, 2= >10% HLS, 1= <10% HLS and 0= no HLS on the back and abdomen. To score the degree of paralysis, 5= severe paralyzed front and rear limbs with no movement, 4= 2 rear limbs were moderately paralyzed with difficulty in movement, 3= paralyzed rear limbs with bending legs, 2= mildly bending rear limbs, 1= slightly bending rear limbs, 0= no paralyzed limbs with normal movement.

### Statistical analysis

Logrank test was used to analyze the difference in survival rate of drug-treated and non-treated transgenic mice. The unpaired t test with Welch’s correction statistic was used to analyze the difference of the tested gene expression between experimental groups. One-way ANOVA with the Kruskal-Wallis test was used to analyze the difference of the severity of HLP and HLS diseases in transgenic mice. Results are considered statistically significant with a *p* value of <0.05. The symbols * and ** are used to indicate *p* values <0.05 and <0.01, respectively.

## Supporting Information

Figure S1 **The cytotoxicity of GA in RD cells.** RD cells were cultivated in 12-well plate and then treated with the different concentration of GA for 3 or 24 hours. The cell viability was measured by CytoTox 96R Assay kit (Promega, WI, USA). The absorbance at 490nm of vehicle (0.1% DMSO)-treated cells was set to 100%. Values represent the mean of three independent experiments, and error bars show the standard deviation of the mean. The concentration of GA that induced 50% of cell death (IC50) after 3 and 24 hours incubation was calculated as 49.6 µM and 40.1 µM, respectively.
(TIF)

Figure S2 **GA downregulates endogenous HER2 expression.** RD cells were treated with 2 µM (dotted curve) or 0 µM (0.1% DMSO, solid curve) of GA for one hour at 37°C. The cells were washed and fixed by 2% paraformaldehyde in PBS for 30 min at room temperature. After washing and permearlizing, the cells were incubated with 1:250 diluted anti-HER-2 antibody at room temperature for one hour. Cells incubated with internal control rabbit IgG (gray zone) were included. Cells were washed and incubated with 1:400 diluted anti-rabbit IgG-TR antibody for another one hour. Stained cells were run on a FACScan and analyzed by using CellQuest software.
(TIF)

Figure S3 **17-AAG cannot resist against CVA16 infection in hSCARB2-transgenic mice.** Seven-day old mice subcutaneously preinfected with 3x10^6^ pfu of CVA16 or the same volume of vehicle (0.1% DMSO plus 5% glucose) were intraperitoneally given 2 µg of 17-AAG twice at the time points of 4 and 24 hours post infection. Mice were monitored daily and survival rates were recorded. Each group consisted of 8 mice and the results were representative of 2 independent experiments. The Logrank test was used for statistical analysis.
(TIF)
