# Supplementary figures and images for: Heat Shock protein 90: Role in Enterovirus 71 Entry and Assembly and Potential Target for Therapy
Source: PLoS One. 2013 Oct 2;8(10):e77133. doi: 10.1371/journal.pone.0077133 (PMC3788750; doi:10.1371/journal.pone.0077133)

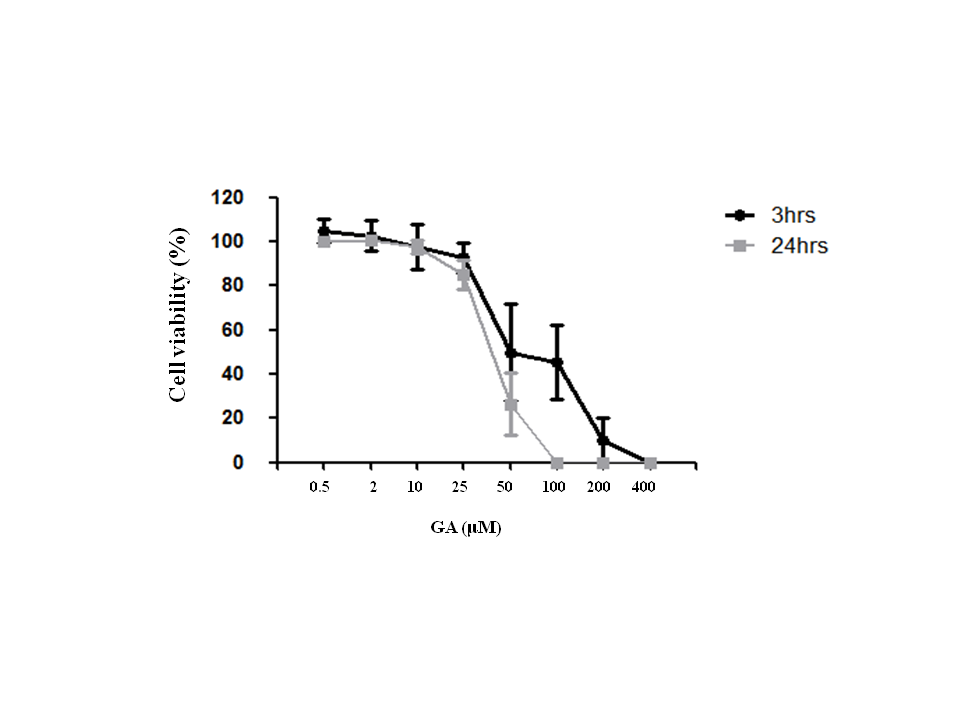

Supplement: Figure S1 — The cytotoxicity of GA in RD cells. RD cells were cultivated in 12-well plate and then treated with the different concentration of GA for 3 or 24 hours. The cell viability was measured by CytoTox 96R Assay kit (Promega, WI, USA). The absorbance at 490nm of vehicle (0.1% DMSO)-treated cells was set to 100%. Values represent the mean of three independent experiments, and error bars show the standard deviation of the mean. The concentration of GA that induced 50% of cell death (IC50) after 3 and 24 hours incubation was calculated as 49.6 µM and 40.1 µM, respectively. (TIF) [file pone.0077133.s001.tif]

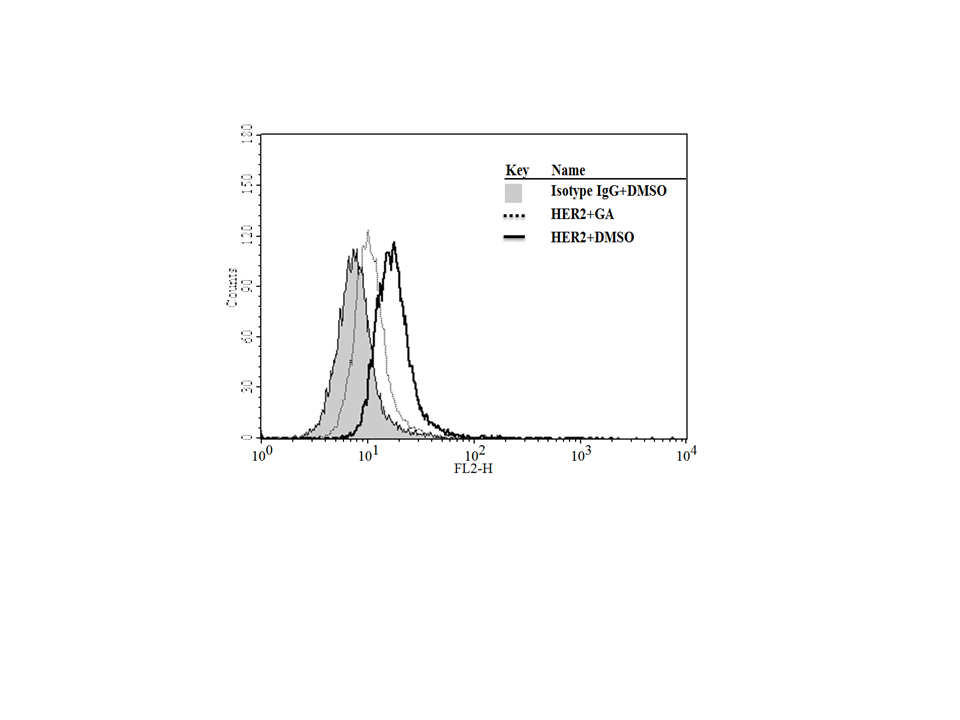

Supplement: Figure S2 — GA downregulates endogenous HER2 expression. RD cells were treated with 2 µM (dotted curve) or 0 µM (0.1% DMSO, solid curve) of GA for one hour at 37°C. The cells were washed and fixed by 2% paraformaldehyde in PBS for 30 min at room temperature. After washing and permearlizing, the cells were incubated with 1:250 diluted anti-HER-2 antibody at room temperature for one hour. Cells incubated with internal control rabbit IgG (gray zone) were included. Cells were washed and incubated with 1:400 diluted anti-rabbit IgG-TR antibody for another one hour. Stained cells were run on a FACScan and analyzed by using CellQuest software. (TIF) [file pone.0077133.s002.tif]

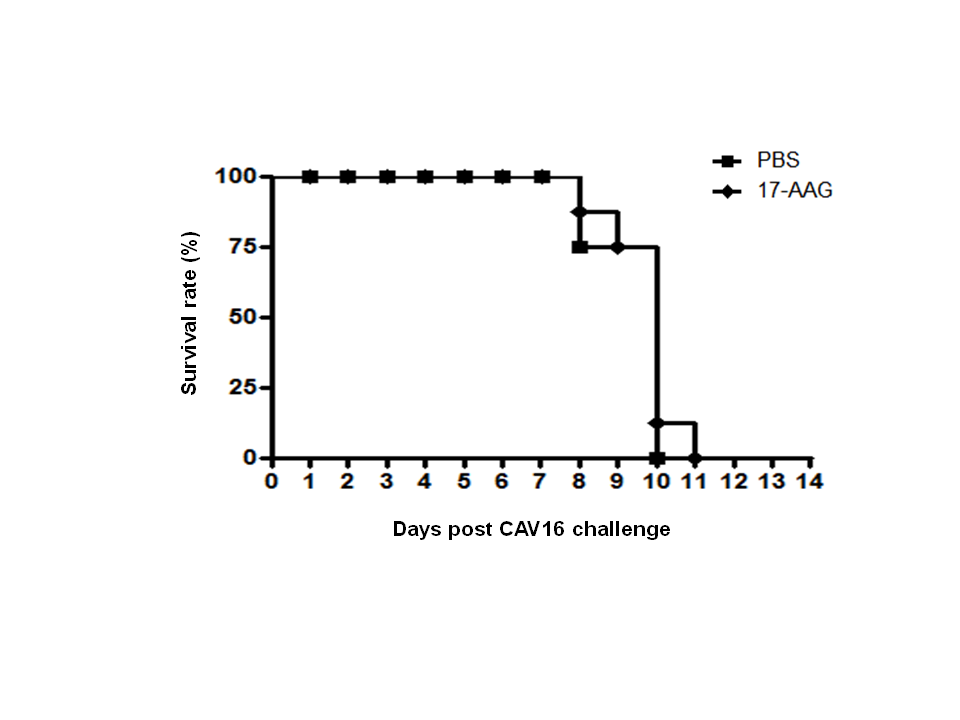

Supplement: Figure S3 — 17-AAG cannot resist against CVA16 infection in hSCARB2-transgenic mice. Seven-day old mice subcutaneously preinfected with 3x10^6^ pfu of CVA16 or the same volume of vehicle (0.1% DMSO plus 5% glucose) were intraperitoneally given 2 µg of 17-AAG twice at the time points of 4 and 24 hours post infection. Mice were monitored daily and survival rates were recorded. Each group consisted of 8 mice and the results were representative of 2 independent experiments. The Logrank test was used for statistical analysis. (TIF) [file pone.0077133.s003.tif]
